# The Effect of Apalutamide on Thyroid Function in Prostate Cancer Patients

**DOI:** 10.1210/jendso/bvae105

**Published:** 2024-05-27

**Authors:** Clare Moffatt, Melissa G Lechner, Trevor E Angell, John Shen, Alexandra Drakaki, Gonzalo J Acosta, Tom Z Liang, Karen Tsai

**Affiliations:** Division of Endocrinology, University of California, Los Angeles (UCLA) Geffen School of Medicine, Los Angeles, CA 90095, USA; Division of Endocrinology, University of California, Los Angeles (UCLA) Geffen School of Medicine, Los Angeles, CA 90095, USA; Division of Endocrinology, University of Southern California, Keck School of Medicine, Los Angeles, CA 90033, USA; Division of Hematology and Oncology, Department of Medicine, University of California, Los Angeles (UCLA) Geffen School of Medicine, Los Angeles, CA 90095, USA; Division of Hematology and Oncology, Department of Medicine, University of California, Los Angeles (UCLA) Geffen School of Medicine, Los Angeles, CA 90095, USA; Division of Endocrinology, Diabetes and Metabolism, University of Florida, Gainesville, FL 32610, USA; Department of Pathology, University of Southern California, Keck School of Medicine, Los Angeles, CA 90033, USA; Department of Diabetes, Endocrinology and Metabolism, City of Hope Comprehensive Cancer Center, Duarte, CA 91010, USA

**Keywords:** apalutamide, thyroid dysfunction, thyroid function tests, hypothyroidism, levothyroxine, thyroid hormone replacement

## Abstract

**Context:**

Apalutamide (APT) is a nonsteroidal antiandrogen medication used to treat metastatic castrate-sensitive and nonmetastatic castrate-resistant prostate cancer. Early clinical trials of APT identified thyroid dysfunction as a common adverse effect of therapy, but the clinical presentation and management of APT-induced hypothyroidism has not been studied.

**Objective:**

The objective of our study is to elucidate the clinical presentation and treatment approach of APT-associated thyroid dysfunction in prostate cancer patients.

**Methods:**

We report a case series of 16 patients with APT-associated thyroid dysfunction during prostate cancer treatment at 2 academic medical centers. Patient clinical parameters, thyroid function laboratory data, and thyroid hormone requirements over the course of APT treatment were analyzed.

**Results:**

Among the 16 patients in our case series with APT-associated hypothyroidism, 3 had no prior thyroid disease and 13 had preexisting hypothyroidism. The patterns of thyroid dysfunction included overt and subclinical hypothyroidism. The median time from APT initiation to thyroid function test abnormality was 19 weeks, but occurred in some cases as early as 2 to 4 weeks. Hypothyroidism was effectively managed with thyroid hormone replacement using levothyroxine (LT4), though some patients with preexisting hypothyroidism required a 2- to 3-fold dose increase while on APT to achieve a euthyroid state. In the subset of patients who completed or stopped APT therapy, thyrotropin levels fell at a median of 11 weeks post APT therapy and thyroid hormone requirements decreased to near pre-APT levels.

**Conclusion:**

APT-associated thyroid dysfunction presents as new or worsening hypothyroidism and should prompt initiation or increase in thyroid hormone replacement. Monitoring of thyroid function tests is recommended every 1 to 2 months for all patients on APT and 2 to 3 months after completion of APT.

Prostate cancer is the second most common cancer type among US adults, with approximately 12.9% of men expected to be diagnosed in their lifetime [1]. Standard treatment can include surgery, radiation therapy, antiandrogen therapy, and chemotherapy. Apalutamide (APT) is an oral, nonsteroidal antiandrogen medication recently approved for the treatment of nonmetastatic castration-resistant (nmCRPC) and metastatic castration-sensitive (mCSPC) prostate cancers following the SPARTAN [2] and TITAN [3] phase 3, double-blind, placebo-controlled trials that showed improved overall survival and metastasis-free survival in these groups.

APT binds directly to the androgen receptor and inhibits the transcriptional activity of the androgen receptor in prostate cancer cells [4]. Thyroid dysfunction was reported as an adverse event in the SPARTAN and TITAN trials, including the development of hypothyroidism in 6.5% to 9.8% of patients as detected by elevated thyrotropin (TSH) levels while on therapy [2, 3]. However, data on the clinical presentation and management of thyroid dysfunction in APT-treated patients remain limited. Specifically, there is uncertainty about the timing of new or worsening hypothyroidism, thyroid hormone replacement dosing needed to achieve euthyroid status, and the duration of delayed effects of APT on thyroid function after completion of therapy. Here we present a case series of 16 patients with APT-associated primary hypothyroidism to elucidate the clinical presentation and treatment approach for this emerging cause of worsening hypothyroidism.

## Materials and Methods

### Study Design and Participants

We report a case series of 16 adult male patients from University of California Los Angeles (UCLA) and City of Hope Comprehensive Cancer Center (COH) with prostate cancer who presented with hypothyroidism while receiving APT therapy. Cases were initially identified by local oncologists or endocrinologists at each site between 2017 and 2022. Each participating institution obtained local institutional review board (IRB) approval or exemption for reporting of these cases (UCLA IRB exemption, City of Hope IRB exemption). Cases were confirmed by manual chart review by board-certified endocrinologists, and deidentified data were collected using a standardized data collection form. Data on patient demographics, oncology history, and comorbid disease were extracted by manual chart review. TSH, free thyroxine (FT4) measurements, and thyroid hormone replacement (all patients received levothyroxine [LT4] only) dosing when pertinent were obtained for each patient prior to, during, and 12 months after APT treatment. While on APT, the highest LT4 doses needed to maintain a euthyroid state during APT treatment after detection of thyroid abnormalities were recorded. Similarly, after APT, all LT4 doses retrieved were the highest LT4 doses within the 1 year after APT cessation needed to maintain a euthyroid state. During APT treatment, the highest “peak” recorded TSH and concurrent FT4 values were also gathered. Weight-based thyroid hormone replacement doses (mcg/kg/day) were calculated prior to, during, and after APT treatment.

### Study Definitions

Hypothyroidism was defined as elevated TSH and low FT4, a TSH level greater than 10mIU/L, or requirement for thyroid hormone replacement. Subclinical hypothyroidism (SCHypo) was defined as elevated TSH with normal FT4 level. Subclinical hyperthyroidism (SCHyper) was defined as low TSH with normal FT4 level, and hyperthyroidism was defined as low TSH with elevated FT4 levels, but neither were detected in any cases. Worsening hypothyroidism was defined as needing an increase in thyroid hormone therapy (eg, LT4) dose to achieve a euthyroid status or an increase in TSH level on current dose of LT4 during APT treatment. Patients who had SCHypo or worsening hypothyroidism but were never started on LT4 or never achieved a euthyroid state were denoted as such. In patients who were started on LT4 or had adjustments in LT4, a euthyroid state was defined as normal TSH or clear documentation in the medical chart that the patient was euthyroid on their LT4 dose.

### Statistical Analyses

Descriptive statistics were used to summarize patient baseline characteristics, initial thyroid function changes, and LT4 dosing before, during, and after APT. Median and interquartile range (IQR) were used as data were not assumed to be normally distributed. Comparisons of TSH and LT4 doses across treatment course among patients were compared using a mixed effects model followed by pairwise comparisons. Statistical analyses and figures were prepared using GraphPad Prism v10 software.

## Results

### Study Population and Baseline Characteristics

Cases (n = 16) were all male patients treated with APT for prostate cancer, with a median age of 66.5 years (IQR, 64.25-71.75 years) (Table 1). Most patients (n = 13, 81.25%) were White and non-Hispanic (n = 15, 93.75%). Three patients (18.75%) had no prior hypothyroidism and 13 (81.25%) had previous hypothyroidism prior to APT treatment. All patients with available Gleason scores had a prostate cancer Gleason score of 7 or greater, and most (n = 13, 81.25%) were classified as high risk of 5-year treatment failure [5]. Clinical response to APT was evenly distributed between partial (n = 6, 37.50%), complete (n = 5, 31.25%), cancer progression (n = 4, 25.00%), and unknown (n = 1, 6.25%). At 1-year follow-up, 4 patients (25.0%) were deceased. The median follow-up time was 27 months (IQR, 22-32.5 months). Six patients (37.5%) received endocrinology consultation or care.

**Table 1.** Patient characteristics for total study population

	Overall (n = 16)
Age at prostate cancer diagnosis, y	
Median (IQR)	66.50 (64.25-71.75)
Race, n (%)	
White	13 (81.25%)
Black	0 (0.00%)
Asian	2 (12.50%)
Other	0 (0.00%)
Unknown	1 (6.25%)
Hispanic, n (%)	
No	15 (93.75%)
Yes	0 (0.00%)
Unknown	1 (6.25%)
Prior hypothyroidism, n (%)	
No	3 (18.75%)
Yes	13 (81.25%)
Gleason score, n (%)	
<7	0 (0.00%)
7	5 (31.25%)
>7	10 (62.50%)
Unknown	1 (6.25%)
D’Amico risk stratification for 5-y treatment failure, n (%)	
Low	0 (0.00%)
Intermediate	3 (18.75%)
High	13 (81.25%)
Best apalutamide clinical response, n (%)	
Partial	6 (37.50%)
Complete	5 (31.25%)
Progression	4 (25.00%)
Unknown	1 (6.25%)
Deceased at time of follow-up, n (%)	
No	12 (75.00%)
Yes	4 (25.00%)
Endocrine follow-up	
No	8 (50.00%)
Yes	6 (37.50%)
Unknown	2 (12.50%)
Follow-up time, mo	
Median (IQR)	27.00 (22.00-32.50)

Abbreviation: IQR, interquartile range.

#### Incidence and pattern of thyroid function abnormalities during apalutamide therapy

To better analyze thyroid function test patterns and thyroid hormone replacement, patients were stratified into groups of either “no prior thyroid disease” (n = 3) or “preexisting hypothyroidism” (n = 13) for subsequent analyses.

### No Prior Thyroid Disease Group

**Figure 1.**
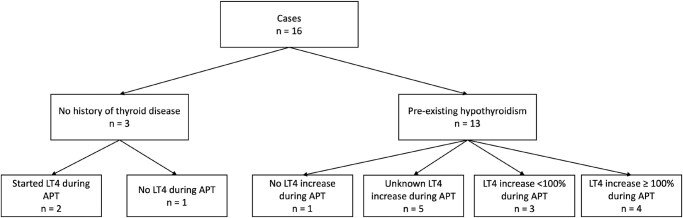
Flowchart of cases by baseline thyroid status and changes in thyroid hormone therapy. APT, apalutamide.

**Figure 2.**
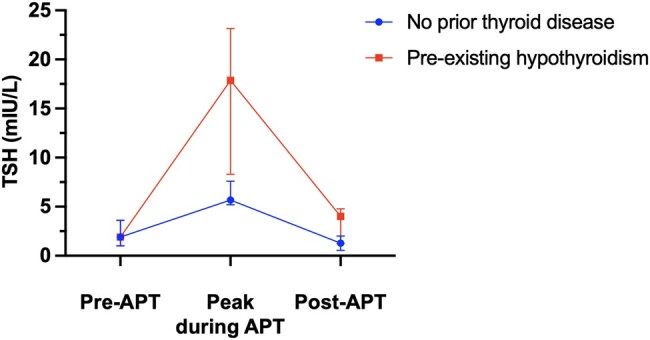
Trends in median thyrotropin (TSH) in patients with and without previous hypothyroidism. Changes in serum TSH levels over the apalutamide (APT) treatment course in patients with no prior thyroid disease (n = 3 for Pre-APT and during APT; n = 2 for Post-APT) and preexisting hypothyroidism (n = 10 for Pre-APT and during APT; n = 8 for Post-APT). Median shown with interquartile range.

Of the 16 patients in our case series, 3 had no history of thyroid disease and presented with incident subclinical hypothyroidism (Fig. 1). In this group, the median baseline TSH was 1.90 mIU/L (IQR, 1.00-3.62 mIU/L) and increased to a median of 5.68 mIU/L (IQR, 5.20-7.60 mIU/L) during APT treatment (3.0-fold increase from baseline) (Table 2 and Fig. 2). Only 2 patients had documented FT4 levels; both were 1.00 ng/mL at time of peak TSH. The median onset of the first TSH abnormality from the start of APT was 19.00 weeks (IQR, 4.00-83.57 weeks) (see Table 2).

**Table 2.** Effect of apalutamide on thyroid function tests in patients with and without prior hypothyroidism

	No prior thyroid disease (N = 3)	Preexisting hypothyroidism (N = 13)
Baseline		
Thyroid function tests		
** **TSH, median (IQR)	1.90 (1.00-3.62)	1.16 (0.51-1.68)*^a^*
** **FT4, median (IQR)	No data	1.27 (1.16-1.40)*^b^*
LT4 dose		
** **LT4, median (IQR)	0.00 (0.00-0.00)	1.14 (0.36-1.46)
During APT therapy		
Time from APT to initial thyroid		
Abnormality, wk		
** **Median (IQR)	19.00 (4.00-83.57)	18.43 (3.93-29.00)*^c^*
Peak abnormality during APT		
** **TSH, median (IQR)	5.68 (5.20-7.60)	17.87 (8.30-23.15)*^a^*
** **FT4, median (IQR)	1.00 (1.00-1.00)*^d^*	1.00 (0.76-1.15)*^c^*

Abbreviations: APT, apalutamide; FT4, free thyroxine (ng/mL); IQR, interquartile range; LT4, levothyroxine (mcg/kg/day); TSH, thyrotropin (mIU/L).

^*a*^n = 10 individuals, *^b^*n = 6 individuals, *^c^*n = 9 individuals, *^d^*n = 2 individuals.

### Preexisting Hypothyroidism Group

Thirteen patients in our case series had a prior history of hypothyroidism and were receiving thyroid hormone replacement. While on APT, 12 patients had worsened hypothyroidism and 1 patient had an increase in TSH but no change in LT4 dose during APT treatment (see Fig. 1). In this group of previously hypothyroid patients, the median baseline TSH pre-APT was 1.16 mIU/L (IQR, 0.51-1.68 mIU/L) and peaked to a median of 17.87 mIU/L (IQR, 8.30-23.15 mIU/L) during APT treatment (15.4-fold increase from baseline) (see Table 2 and Fig. 2). The median onset of the first TSH abnormality from the start of APT was 18.43 weeks (IQR, 3.93-29.00 weeks). Among the 9 patients with documented FT4 levels while on APT, the median FT4 was 1.00 ng/mL (IQR, 0.76-1.15 ng/mL).

#### Thyroid hormone medication changes during apalutamide therapy

##### No prior thyroid disease group

Of the 3 patients with incident subclinical hypothyroidism, 2 were started on LT4 during APT treatment. The median LT4 dose was 0.94 mcg/kg/day (IQR, 0.42-1.45 mcg/kg/day) (Table 3). Both patients had normalization of their TSH values to the reference range on LT4. The third patient did not receive thyroid hormone replacement therapy.

**Table 3.** Levothyroxine replacement dose in patients who achieved euthyroid state on apalutamide

	No prior thyroid disease (N = 3)	Preexisting hypothyroidism (N = 8)
Best thyroid function tests during APT		
TSH, median (IQR)	4.50 (2.60-5.12)	3.39 (1.35-5.17)*^b^*
FT4, median (IQR)	1.20 (1.20-1.20)*^a^*	1.31 (1.00-1.66)*^c^*
LT4 requirement during APT		
Dose, median (IQR)	0.94 (0.42-1.45)*^a^*	1.82 (0.60-3.08)

Abbreviations: APT, apalutamide; FT4, free thyroxine (ng/mL); IQR, interquartile range; LT4, levothyroxine (mcg/kg/day); TSH, thyrotropin (mIU/L).

^*a*^n = 2 individuals, *^b^*n = 5 individuals, ^*c*^n = 4 individuals.

##### Preexisting hypothyroidism group

Of the 13 patients with prior hypothyroidism, 12 patients had a dose increase of LT4 while on APT in response to worsening hypothyroidism on laboratory test evaluation (rise in TSH), while 1 patient received no dose increase despite laboratory values showing subclinical hypothyroidism (see Fig. 1). The median LT4 requirement prior to APT among all 13 patients was 1.14 mcg/kg/day (IQR, 0.36-1.46 mcg/kg/day) (see Table 2). Among the 8 patients who achieved a euthyroid state while on APT and for whom thyroid hormone dose information (mcg/kg/day) was available, the median LT4 dose on APT was 1.82 mcg/kg/day (IQR, 0.60-3.08 mcg/kg/day) (see Table 3). Individual patient dose increases ranged from 20% to 222% [6]. In summary, patients required higher thyroid hormone replacement while on APT therapy, with some requiring a 2- to 3-fold increase to achieve a euthyroid state.

#### Thyroid function tests and thyroid hormone medication changes after apalutamide therapy

##### No prior thyroid disease group

Among the group of patients with no prior thyroid disease, 2 completed APT treatment and 1 remained on APT at the end of data collection. In the 2 patients who stopped APT, TSH levels decreased from a median of 5.68 mIU/L on therapy (IQR, 5.20-7.60 mIU/L) (see Table 2 and Fig. 2) to 1.28 mIU/L (IQR, 0.55-2.00 mIU/L) after APT. The median time from APT discontinuation to the first documented decrease in TSH levels was 10.43 weeks (IQR, 7.71-13.14 weeks). One patient continued to require LT4 therapy, but at a lower dose—requiring 1.45 mcg/kg/day to achieve a euthyroid state while on APT and only 1.19 mcg/kg/day after cessation of APT. The other patient developed subclinical hypothyroidism while on APT but was not treated with LT4. However, after APT therapy, thyroid function tests reverted to the reference range and the patient did not require thyroid hormone replacement.

##### Preexisting hypothyroidism group

**Figure 3.**
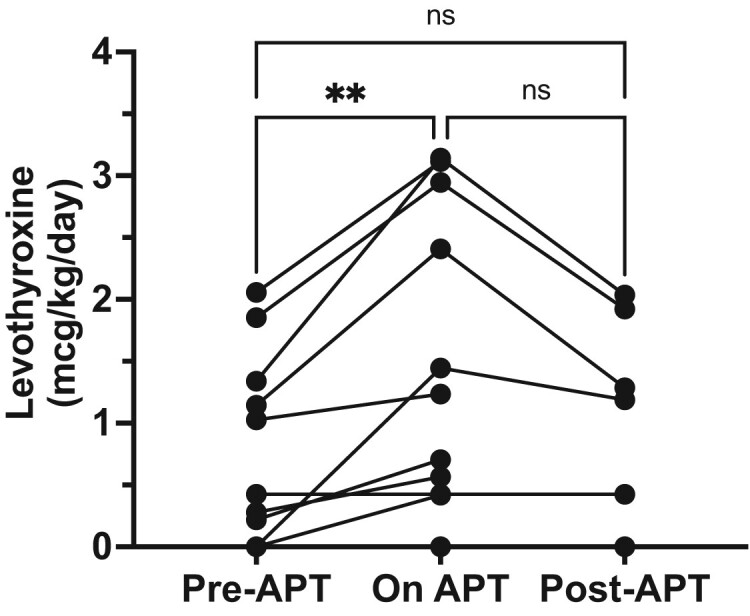
Changes in thyroid hormone dose over the treatment course in patients who achieved euthyroid state while on apalutamide (APT) therapy. All patients were treated with levothyroxine. Each dot and line represents an individual patient over time. Mixed effects analysis of paired data *P* = .0188, followed by subsequent pairwise comparisons between all groups. **P less than .01.

In the group of patients with preexisting hypothyroidism, the median TSH also fell following discontinuation of APT. The median peak TSH was 17.87 mIU/L (IQR, 8.30-23.15) on APT (see Table 2) compared to 4.00 mIU/L (IQR, 1.23-4.78 mIU/L) after APT (see Fig. 2). The median time from APT discontinuation to the first recorded decrease in TSH level was 11.71 weeks (IQR, 4.79-26.00 weeks). In the 8 patients who were euthyroid post-APT, the median LT4 post-APT dose remained slightly elevated at 1.29 mcg/kg/day (IQR, 1.11-1.79 mcg/kg/day) after cessation of APT compared to the median pre-APT LT4 dose of 1.14 mcg/kg/day (IQR, 0.36-1.46 mcg/kg/day) (see Table 2 and Fig. 3). Fig. 3 outlines the trajectory of each patient's LT4 adjustments before, during, and after APT, demonstrating overall the increase in LT4 dose from baseline while on APT and their decrease back to near baseline after APT.

## Discussion

Our case series of 16 patients highlights the clinical presentation and outcome of an emerging cause of cancer treatment-associated thyroid dysfunction. Thyroid dysfunction is a common adverse event during APT therapy in prostate cancer patients for which clinical guidance on the optimal timing of laboratory monitoring and thyroid hormone replacement is currently lacking. Patients with no prior thyroid disease and with preexisting hypothyroidism both demonstrated abnormal TSH elevations consistent with new or worsening subclinical hypothyroidism, indicating broad susceptibility to this side effect. In addition, while hypothyroidism is more common in women, these data demonstrate that thyroid function abnormalities should not be neglected in this male patient population.

The calculated median time from the start of APT to first thyroid abnormality in all patients with or without a prior history of hypothyroidism was approximately 19 weeks. However, thyroid abnormalities were detected in patients in both groups as early as 2 to 4 weeks, which is much earlier than the recommended 4-month interval thyroid function test monitoring seen in the SPARTAN and TITAN clinical trials [2, 3]. Specifically, based on these data, we recommend that patients have pre-APT baseline thyroid function testing and thereafter every 1 to 2 months during APT treatment to detect thyroid abnormalities and facilitate treatment with thyroid hormone replacement.

Thyroid hormone replacement with LT4 proved effective in restoring thyroid hormone balance to euthyroid status (as determined by laboratory monitoring). Notably, patients with preexisting hypothyroidism required an increase in LT4 dose from a median of 1.14 to 1.82 mcg/kg/day, representing a 2- to 3-fold dose escalation in some patients. Furthermore, the median dose required to achieve a normal TSH in patients with preexisting hypothyroidism was near the 1.6 mcg/kg/day dose recommended by the American Thyroid Association for athyreotic patients [7] and guidance from another report on hypothyroid patients on APT [8]. Therefore, clinicians should be prepared to augment thyroid hormone replacement doses as needed to normalize TSH levels in these patients. More conservative dose increases in LT4 should be considered in men with preexisting hypothyroidism on APT who have higher overall weights but a body composition of mostly fat instead of muscle.

While the mechanism of thyroid dysfunction during APT therapy remains unclear, one prior study suggested that APT led to greater T4 metabolism and clearance via increased hepatic UDP-glucuronosyltransferase activity [9]. This mechanism is consistent with the observed increased need for T4 during APT therapy and subsequent return to near baseline after cessation of APT therapy in most patients in our series. The reason the return to lower thyroid hormone doses took up to several months in some patients may not be fully explained by this hypothesis, and additional mechanistic studies are needed.

Limitations of our study include its small sample size and the inherent retrospective nature of a case series. Although we were able to find singular TSH values before, during, and after treatment for many patients, prospective assessment of thyroid function at regular intervals is needed to more completely capture the dynamics of thyroid function changes in patients on APT therapy. In addition, thyroxine-binding globulin testing was available for only a minority of patients (with normal results) and this additional sex-hormone dependent variable should be evaluated in future studies. Other remaining questions include evaluating the risk factors that may predispose some patients to develop new or worsening hypothyroidism and the relationship between thyroid function abnormalities and clinical response to APT, such as overall survival or progression-free survival. Finally, our study highlights the importance of early multidisciplinary care between endocrinology and oncology when thyroid function abnormalities are seen during APT to address new or worsening hypothyroidism. In patients where frequent thyroid function monitoring may not be feasible or endocrinology care is not available or readily accessible, other alternative medications such as darolutamide or enzalutamide may be considered.

## Conclusion

APT is an effective treatment for nmCRPC and mCSPC, but it can cause new or worsening hypothyroidism. TSH elevations during APT treatment are commonly encountered, perhaps more noticeably in patients with preexisting hypothyroidism, and should not be ignored. Often, patients will require initiation or adjustment of thyroid hormone replacement during APT treatment. When on APT, thyroid function should be monitored approximately every 1 to 2 months to allow for the timely detection of thyroid function abnormalities. Although TSH appears to begin to decrease as early as within several weeks after cessation of APT, it is recommended to check thyroid function tests 2 to 3 months after APT discontinuation due to a possible decrease in LT4 dose toward baseline pre-APT levels. These data provide improved guidance to clinicians for the monitoring of thyroid function in patients receiving APT therapy.

## Data Availability

Original data generated and analyzed during this study are included in this published article or in the data repositories listed in “References.”

## Abbreviations

APT: apalutamide
COH: City of Hope Comprehensive Cancer Center
FT4: free thyroxine
IQR: interquartile range
LT4: levothyroxine
nmCRPC: nonmetastatic castration-resistant prostate cancer
mCSPC: metastatic castration-sensitive prostate cancer
SCHyper: subclinical hyperthyroidism
SCHypo: subclinical hypothyroidism
TSH: thyrotropin
UCLA: University of California Los Angeles
